# Cellular processes driving gastrulation in the avian embryo

**DOI:** 10.1016/j.mod.2020.103624

**Published:** 2020-09

**Authors:** Guillermo Serrano Nájera, Cornelis J. Weijer

**Affiliations:** Division of Cell and Developmental Biology, School of Life Sciences, University of Dundee, Dundee DD1 5EH, UK

**Keywords:** Gastrulation, Chick embryo, Morphogenesis, Cell flows, Intercalation, Patterning

## Abstract

Gastrulation consists in the dramatic reorganisation of the epiblast, a one-cell thick epithelial sheet, into a multilayered embryo. In chick, the formation of the internal layers requires the generation of a macroscopic convection-like flow, which involves up to 50,000 epithelial cells in the epiblast. These cell movements locate the mesendoderm precursors into the midline of the epiblast to form the primitive streak. There they acquire a mesenchymal phenotype, ingress into the embryo and migrate outward to populate the inner embryonic layers. This review covers what is currently understood about how cell behaviours ultimately cause these morphogenetic events and how they are regulated. We discuss 1) how the biochemical patterning of the embryo before gastrulation creates compartments of differential cell behaviours, 2) how the global epithelial flows arise from the coordinated actions of individual cells, 3) how the cells delaminate individually from the epiblast during the ingression, and 4) how cells move after the ingression following stereotypical migration routes. We conclude by exploring new technical advances that will facilitate future research in the chick model system.

## 1 Introduction

Gastrulation is an early major morphogenetic event in animal development. In vertebrates, during this process, a pluripotent single-layered epithelial, the epiblast, is restructured into three germ layers, the ectoderm, mesoderm and endoderm with restricted cell fate potentials. As most developmental stages, gastrulation is driven by four critical cell behaviours, i.e. cell division, cell differentiation, cell shape changes and cell movements. Essentially, self-organising cell-cell signalling mechanisms control all these cell behaviours. One of the main goals of developmental biology is to understand how the complex spatio-temporal dynamic interactions between signalling and cell behaviours ultimately control the assembly of initially hundreds to thousands differentiating cells into increasingly elaborated tissues and organs making up the embryo.

The chick is a widely used amniote model system to study the cellular mechanics of gastrulation. The early chick embryo has two significant advantages as a developmental model: 1) it can be easily cultivated *ex-ovo* for extended periods of time making it accessible to manipulation and 2) its flat morphology and robustness facilitates grafting and transplantation experiments and simplifies long-term live imaging.

In this review, we aim to present our current understanding of the integration of fundamental cellular processes during gastrulation in the chick embryo. The embryonic area of avian embryos before gastrulation is essentially a flat disk of cells divided into two layers: the upper epiblast epithelial layer and the lower hypoblast. The next major event is the induction of the mesendoderm in a sickle shape domain at the posterior interface between the embryonic and extraembryonic tissues. The coordinated movement of thousands of cells organised in two counter-rotating vortex flows of millimetre scale in the epiblast transports these mesendoderm precursors to the midline to form the primitive streak, where they ingress. Finally, after ingression, cells migrate laterally to form the mesoderm and endoderm.

In this review we briefly review tissue organisation and pre-patterning of the early chick embryo, the mechanisms implied in setting up the anterior-posterior axis and the induction of the mesendoderm precursors. Next, we explore how after the induction of the mesendoderm individual cell behaviours give rise to the epiblast flows and how they might be coordinated. Later we examine the process of individual cell ingressions through the primitive streak. We finish with a discussion of the mode of migration of mesoderm cells after ingression and the signalling mechanisms known to contribute to the guidance of their migratory pathways.

Each section starts by summarising the essential organisation of the tissue and the cell behaviours associated with the process, followed by a discussion of the mechanisms driving cell and tissue behaviours, and a revision of the current understanding of signalling mechanisms that have been implicated in the control and integration of these individual cellular activities. We conclude with an outlook, including a brief overview of exciting new technologies that are expected to enhance the avian model.

## 2 Patterning before gastrulation

There are three consecutive essential events in the early patterning of the avian embryo: 1) the embryonic and extraembryonic territories segregate into radially symmetric territories, 2) the determination of the anterior-posterior polarity of the embryo, 3) the patterning of the epiblast by the induction of mesendoderm in the sickle-shaped posterior domain of the embryo.

The first two events, the separation of the embryonic and extraembryonic tissues and setting up of the anterior-posterior polarity, occur before the egg is laid and are less accessible to experimentation. Here, we summarise some key findings on the determination of the avian embryonic territories and their patterning before the onset of gastrulation. For more extensive reviews on this topic see (Raffaelli and Stern, 2019; Sheng, 2014).

### 2.1 Segregation of embryonic and extra embryonic territories

**Fig. 1:**
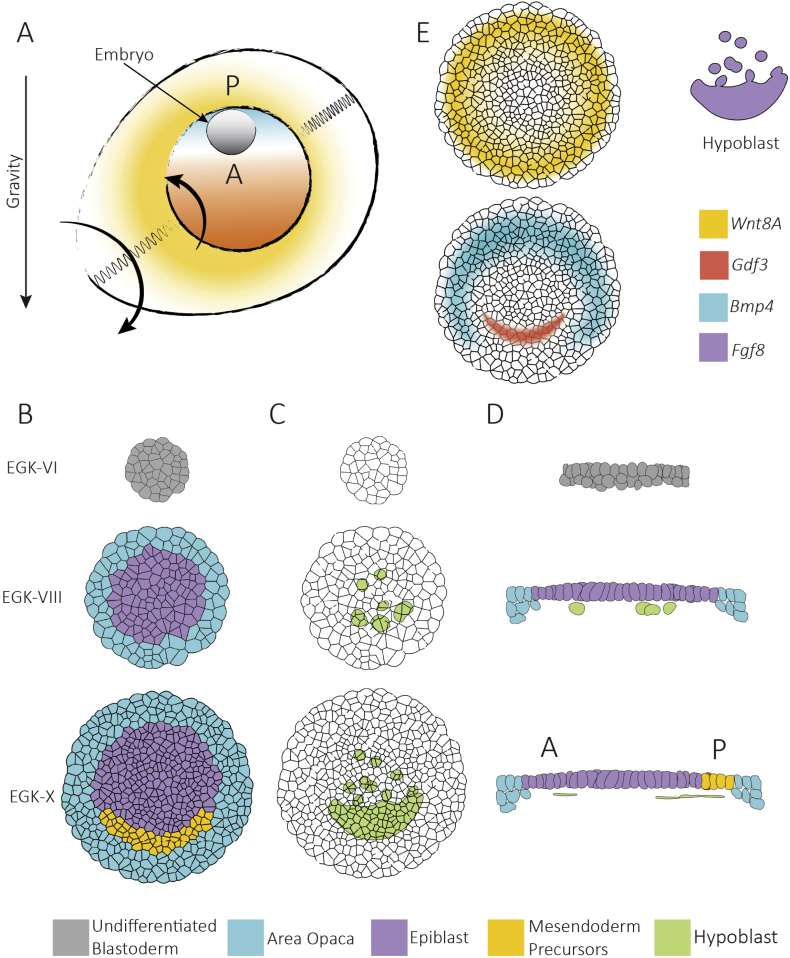
Avian development before gastrulation. A) Orientation of the embryo on the yolk during intrauterine stages of development. Arrows indicate the direction of rotation of the egg and yolk during the transition through the oviduct. B) Segregation of the embryonic epiblast and extra embryonic Area Opaca. C) Development of the hypoblast. D) Cross sections during intrauterine development. E) Spatial patterning of essential paracrine developmental signals: *Wnt8A* is initially expressed in the Area Opaca, there is an anterior-posterior gradient of *Bmp4* in the Area Opaca, *Gdf3* is expressed in the posterior epiblast and *Fgf8* is produced in the hypoblast.

The chick embryo is situated on top of the yolk under the vitelline membrane (Fig. 1A). After a period of rapid cleavages, during the transition of the egg through the oviduct, the chick embryo initially forms a multi-layered disk of epithelial cells during the first 10 h of intrauterine development.

The cells are open to the yolk during the initial cleavage divisions. However, around stage EGK III they start to close and a fluid filled cavity separating the cells from the yolk, the subgerminal cavity, forms. In the following 10–12 h of uterine development, the radial embryonic and extraembryonic territories differentiate from this stratified epithelium (Fig. 1B–D). The central embryonic area, the *Area Pellucida* (AP), forms a monolayer of epithelial cells (the epiblast or *embryo proper*) via cell shedding and possibly radial intercalation (Eyal-Giladi and Kochav, 1976; Kochav et al., 1980; Sheng, 2014). The remaining outer stratified epithelial ring of tissue, the *Area Opaca* (AO) will generate extraembryonic tissues in conjunction with the extraembryonic mesoderm produced later during gastrulation (Hatada and Stern, 1994; Pasteels, 1940; Spratt Jr., 1946). The AO and AP are separated by a thin strip of epithelial cells, the marginal zone (MZ), which has been proposed to play a crucial role in the onset of gastrulation (Azar and Eyal-Giladi, 1979; Eyal-Giladi and Khaner, 1989; Khaner and Eyalgiladi, 1989).

Lastly, the hypoblast forms as a discontinuous sheet of cells underneath the embryo. The hypoblast forms by the merging of the hypoblast islands, loose clusters of cells under the AP. The hypoblast is formed by polyingression from the epiblast but may also include remnant cells of the layer reduction of the embryonic territory (Eyal-Giladi and Kochav, 1976; Kochav et al., 1980; Sheng, 2014). Hypoblast cells are denser posteriorly, where they begin to fuse into an epithelial monolayer in a posterior to anterior manner creating the hypoblast under the epiblast, but not extending to the marginal zone (Fig. 1C). The posterior hypoblast fuses with and later is displaced by the endoblast a group of cells from the posterior deeper layer underlying the posterior marginal zone (Raffaelli and Stern, 2019; Stern and Canning, 1990; Vakaet, 1962).

Only cells in the outer periphery of the AO attach to the vitelline membrane. These edge cells extend protrusions and crawl continuously outwards, keeping the rest of the embryo under tension (Bellairs et al., 1969; Downie, 1976). The basal cells of the AO are in direct contact with the yolk, while cells in epiblast are separated from the yolk by the subgerminal cavity. At the stage of egg-laying, around 22 h after fertilisation, the embryo contains around 50,000 cells and it is divided into at least four cell populations: the AO, epiblast, MZ and the hypoblast, characterised by specific lineage-markers well described in mammalian models, reviewed in Sheng (2014).

### 2.2 Anterior-posterior axis formation

When the egg is laid, the anterior-posterior polarity of the blastoderm is already established. Von Baer showed that if the sharp-end of the egg is positioned to the right, the embryo usually develops perpendicularly to the long axis with the posterior end closer to the observer (von Baer, 1828). Other observations further support the notion that the symmetry is already broken: the hypoblast is denser under the posterior part of the epiblast, and there is a gradient of regeneration potential along the anterior-posterior axis when the embryo is cut into pieces (Eyal-Giladi and Kochav, 1976; Lutz, 1949; Spratt and Haas, 1960).

In eggs extracted from the hen at intrauterine stages (EGK VI-X) and cultivated in a tilted position, embryos develop along the axis of gravity, where posterior end is always on top (Eyal-Giladi and Fabian, 1980; Kochav and Eyal-Giladi, 1971). In normal development gravity also plays a role in determining the anterior-posterior orientation (Fig. 1A). Due to the complex structure of the yolk and its inherent density gradient, the embryo normally sits on top of the yolk. As part of its intrauterine development, the egg rotates clockwise during soft- and hard-shell production in the shell gland. The density gradient in the yolk prevents its rotation in the egg, and as a consequence, the chalazas (the fibrous material that connect the shell with the yolk) wind up (Clavert, 1962). A slight lag between the egg's rotation and the yolk's restoring counter rotational movements maintain the embryo in a slightly tilted position perpendicular to the long axis of the egg. The uppermost part of the blastoderm acquires a posterior identity explaining von Baer's rule. A likely interpretation is that the slight rotation of the yolk relative to the embryo results in displacement of yet unknown determinants that determine the polarity.

Even though the radial symmetry is broken early in development, the embryo retains a great regulatory potential to regenerate the anterior-posterior polarity: Any slice of the chick blastula can regenerate a whole embryo, as long as it contains part of the MZ and associated deeper cells (Azar and Eyal-Giladi, 1979). The study of *in vitro* amniote model systems such as gastruloids derived from mouse embryonic stem cells (Beccari et al., 2018; Turner et al., 2017; van den Brink et al., 2014) and human embryonic stem cells (Simunovic et al., 2019) have shown that they spontaneously break the anterior-posterior polarity, in the absence of extraembryonic tissues. However, they need the addition of uniform signals like BMP4 or WNT to pattern the anterior-posterior axis. These observations show that the anterior-posterior polarity can also be established in a gravity-independent way.

Recently, a self-organising reaction-diffusion mechanism has been proposed to explain the regeneration of the anterior-posterior axis in chick, which could resolve some of these problems (Arias et al., 2017). In this model, the paracrine signals are locally received, amplified, and relayed by target cells alleviating the requirement for long-range diffusion. The mechanism is based on the observation that GDF3 (chick Vg1), localised in the posterior part of the marginal zone, inhibits BMP4. BMP4 activates the expression of GDF3 at low concentrations, but inhibits it at high concentrations, resulting in a stable exclusion pattern of GDF3 and BMP4. Even if it does not initiate anterior-posterior pattern formation, this, or a similar system, could play a role in stabilisation and maintenance of the pattern during early development. The existence of self-organisation and maintenance systems is shown by the finding that the surgical removal of the hypoblast in chicken or its equivalent in mouse, the Visceral Endoderm results in the generation of multiple primitive streaks in both organisms (Bertocchini and Stern, 2002; Perea-Gomez et al., 2004; Yamamoto et al., 2004). This shows that both in chicken and mouse symmetry-breaking is not robust in the absence of hypoblast/Visceral Endoderm, and that the epiblast has an intrinsic power to generate new body axis.

A possible interpretation is that the hypoblast biases the intrinsic mechanism for symmetry-breaking in the epiblast to create only one axis (Turner et al., 2017). In chick, gravity could condition the formation of the hypoblast during uterine stages, which in turn, could bias an epiblast-intrinsic reaction-diffusion mechanism to generate and/or amplify a single robust signal for anterior-posterior axis determination.

### 2.3 Patterning of the epiblast

As in other organisms, the induction and differentiation of the mesendoderm in a sickle-shaped region at the posterior interface of the AO and AP, are dependent on paracrine signals that derive both from the embryonic tissues as well as the extraembryonic tissues (AO, MZ, and hypoblast) (Fig. 1E).

The essential role of the extraembryonic tissues was first shown by transplantation and dissection experiments, that established that the AO, especially the PMZ, was required for the regeneration of the hypoblast (Azar and Eyal-Giladi, 1979) and the induction of the mesendoderm. A grafted portion of the PMZ to regions adjacent to the MZ can induce the differentiation of the mesoderm and initiate the formation of a PS while preventing the formation of a secondary axis (Eyal-Giladi and Khaner, 1989; Khaner and Eyalgiladi, 1989).

The PMZ is characterised by expression of GDF3, a TGFβ family member. When ectopically expressed close to the MZ, GDF3 can induce the differentiation of epiblast cells into mesendoderm precursors followed by the formation of the PS, suggesting that PMZ is equivalent to the Nieuwkoop centre of amphibians (Bertocchini et al., 2004; Seleiro et al., 1996; Shah et al., 1997; Skromne and Stern, 2002).

The competence of the MZ to respond to GDF3 depends on WNT8A (WNTC8) expression (Hume and Dodd, 1993). Immediately after laying, WNT8A is initially expressed in the AO but later is restricted to the PMZ and the primitive streak as the mesendoderm differentiates (Alev et al., 2013; Hume and Dodd, 1993). Finally, the co-expression of GDF3 and WNT1 can initiate the formation of the PS even far from MZ, suggesting that WNT signalling makes the cells competent for the formation of the PS, while TGFβ signalling works as an inducer (Skromne and Stern, 2001). The PMZ does not contribute to the embryonic tissues, only having a role inducing the mesoderm which arises where a TGFβ and WNT signalling converge (Skromne and Stern, 2001). These findings suggest that the PMZ is analogous to the Nieuwkoop centre of amphibians.

Alternatively, it has been proposed that, only the cells in the Koller's sickle, the inner cells located at a thickening of the tissue in the PMZ, form the Niewkoop centre (Callebaut, 2005; Callebaut and Van Nueten, 1994; Callebaut et al., 2005). Recently, Lee et al. (2020) took a systematic dissection and sequencing approach to define gene expression patterns in the chick gastrula before gastrulation (HH1). They managed to identify sets of transcripts which will help to define embryonic territories such as the AO, MZ, Koller's sickle, and the hypoblast unambiguously. Interestingly, their findings show that although *Gdf3*, *Pitx2* (a regulatory component controlling *Gdf3* expression (Torlopp et al., 2014)) and *Nodal* are all expressed in the PMZ their expression levels are higher in Koller's Sickle, vastly strengthening the notion that it represents the Nieuwkoop centre. Remarkably, they did not identify genes specific to the MZ, which suggests that the MZ is defined by the convergence of signals from the AP and the AO.

As in other systems, there is a prominent role for Fibroblast Growth Factor (FGF) signalling in mesendoderm differentiation. At the early stages of development, FGF4/8 is expressed in the PMZ and the hypoblast; whereas later, FGF4/8 is produced by cells in the streak (Alev et al., 2013; Hardy et al., 2011), which could reflect a possible positive feedback of FGF expression between the epiblast and hypoblast. Notably, the inhibition of FGF signalling prevents the formation of the mesendoderm and blocks gastrulation movements, while ectopic FGF can induce an extra streak (Bertocchini et al., 2004; Chuai et al., 2006; Hardy et al., 2011). Furthermore, the ubiquitous addition of FGF results in the differentiation of a ring of mesendoderm precursors all along the MZ, presumably where FGF coincides with WNT secreted by the AO. Indeed, the application of WNT8A and FGF2,4,8 in the subgerminal space of freshly laid eggs results in the differentiation of all cells in the epiblast into mesendoderm showing that mesendoderm induction requires both FGF and WNT signalling (Alev et al., 2013).

In addition to FGF, the hypoblast produces inhibitory signals which block the formation of the PS. This inhibitory effect of the hypoblast is mediated by production of CER1 (Cerberus), an antagonist of the TGFβ, WNT and BMP signalling pathways (Bertocchini and Stern, 2002). Nodal, a TGFβ family factor can only induce the differentiation of the mesendoderm and the generation of a new axis in the absence of hypoblast (Bertocchini and Stern, 2002). It has been proposed that the displacement of the primary hypoblast by the cells of the endoblast (which do not express CER1) could release the CER1-mediated inhibition of the PS formation (Bertocchini et al., 2004; Bertocchini and Stern, 2002). This way, the hypoblast could control the positioning and timing of the formation of the PS through the secretion of FGFs and inhibitors of TGFβ and WNT signalling.

BMP signalling has a role defining the anterior character in the epiblast (see also Section 2.2). The anterior part of the epiblast and the AO express BMP4/7 (Lee et al., 2020; Streit et al., 1998). The ectopic expression of BMP4/7 in the posterior blastoderm blocks the formation of the PS; while ectopic application of Chordin (BMP antagonist) in the anterior AP can induce a PS (Streit et al., 1998). The anterior part of the epiblast is further characterised by the expression of the transcription factor GATA2. Blocking GATA2 expression can result in the expression of ectopic PS markers and axis formation (Bertocchini and Stern, 2012). The ectopic expression BMP4 upregulates GATA2 (Arias et al., 2017); suggesting that the BMP4-GATA2 axis has a significant role in the determination of the anterior identity of the epiblast, which will give rise to embryonic ectoderm later in development.

Despite substantial progress in understanding the early intrauterine part of early chick embryo development, many detailed mechanistic questions remain. Which signals control the differentiation of embryonic and extraembryonic tissues. What mechanisms result in their spatial separation? What is the molecular mechanism of the establishment of the anterior-posterior polarity in the embryo? In general, it remains to be understood how signals and gene regulatory networks interact to set up the observed spatial distribution of cell types in the early embryo in a quantitative manner.

## 3 Cell and tissue behaviours driving the formation of the primitive streak

Early embryos of birds (chick, quail, duck) and many mammals (pig, cow, sheep, rabbit and humans) are large flat epithelial disks. The patterning of the embryonic, extraembryonic and mesendoderm results in the precise compartmentalisation of cell behaviours. The differential arrangement of behaviours, in turn, generate large-scale flows which transport the mesendoderm precursors to locate the mesendoderm precursors on the midline of the epiblast where they ingress creating the PS.

Paradoxically, avian gastrulation presents more similarities with the mammals than with the other sauropsids (reptiles) probably as a result of convergent evolution (Arendt and Nubler-Jung, 1999; Stower and Bertocchini, 2017). Reptiles, instead, internalise the mesendoderm through the blastoporal canal, where the cells roll-in under the epiblast (amphibian-like involution) in the anterior part, and ingress as individual cells in the posterior part, the primitive plate (similar to the primitive streak in birds and mammals). Nevertheless, at the molecular level, there are many similarities among amniote embryos reviewed in Stower and Bertocchini (2017).

Rodents are a notable exception among mammals, presenting a modified early development: murine embryos are not flat, but cup-shaped and the mesendoderm precursors are specified directly in the area that will constitute the primitive streak, where they ingress as individual cells (Williams et al., 2012). For this reason, the experimentally accessible avian embryos will work as an important alternative to study gastrulation dynamics in the mammals. In this section, we discuss how the epithelial cell movements are generated from the individual cell behaviours, as well as how the cell flows might be coordinated.

### 3.1 The tissue flows driving streak formation

**Fig. 2:**
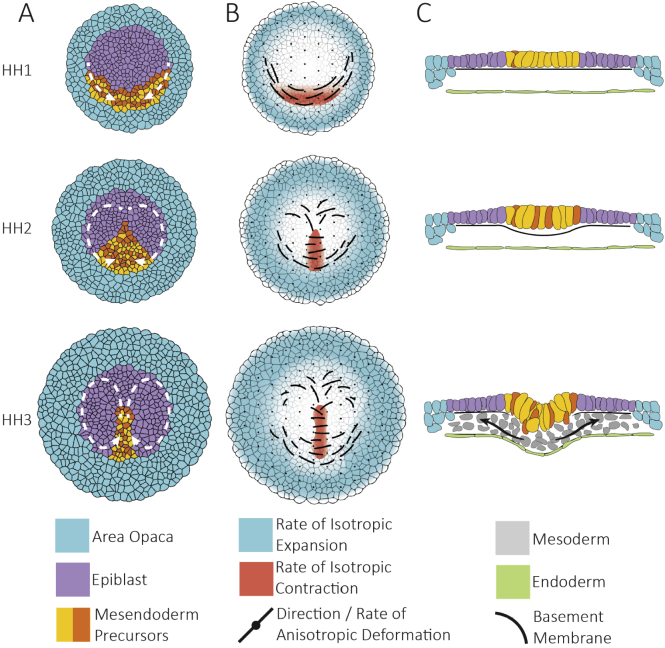
Tissue flows driving streak formation. A) Tissue flow pattern in the embryo (white arrows). Every cell represents ~50 cells in the real embryo. B) Isotropic and anisotropic components of the strain rates derived from the tissue flow. The isotropic component results from cell growth and ingression while the anisotropic component is the product of asymmetric cell rearrangements and cell shape changes. C) Cross sections during streak formation. Cells in the mesendoderm become increasingly columnar as they approach the midline. After digestion of the basement membrane, mesendoderm precursor cells undergo EMT and migrate out to populate the inner embryonic layers.

In avian embryos, a macroscopic convective flow of cells in the epiblast moves the mesendoderm precursors from the posterior sickle-shaped domain into the midline, where they then form the primitive streak (Fig. 2A). These tissue flows are known as Polonaise movements, after the original observations done by time-lapse microphotography of coloured cells (Gräper, 1929; Spratt Jr., 1942; Spratt Jr., 1946; Wetzel, 1929). Since then, the development of fluorescently-labelled transgenic strains has allowed the recording of the cell-flows with great precision: two counter-rotational flows converging towards the posterior midline are contained within the epiblast, while the tissue in the AO expands radially outwards (Rozbicki et al., 2015; Saadaoui et al., 2020).

**Fig. 3:**
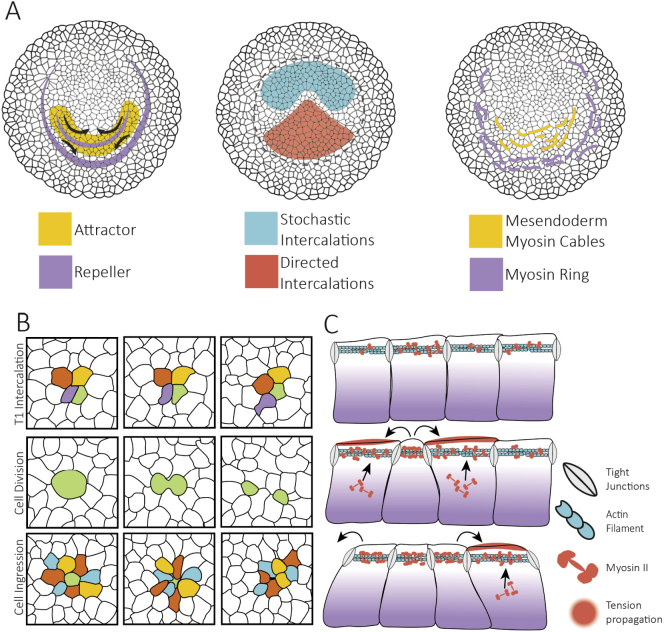
Origins of flow organisation. A) Flow organisers. From left to right: Attractors and repellers of the tissue flow, territories of directed and stochastic cell intercalation and myosin cables B) Cell behaviours that drive epithelial tissue flows, cell intercalation, cell division and ingressions. Division and ingression also contribute to cell rearrangements since they create changes of epithelial topology. C) Mechanism of contraction propagation based on tension dependent stabilisation of apical junctional myosin. From top to bottom: 1) The apical contraction of an individual cell due to a local accumulation of myosin, 2) results in the stretching of neighbouring cell junctions. The increase in tension produced by the stretching leads to the local stabilisation of myosin in these cells resulting in 3) apical contraction in these cells and propagation to further neighbours.

The detailed analysis of the tissue movement velocity fields offers novel insights into the establishment of the flows. Tissue velocity fields can be decomposed into an attracting and two repelling spatiotemporal structures (Serra et al., 2020) (Fig. 3A). The flow attractor maps directly on the mesendoderm tissue that is going to form the streak, while one of the repellers is located at the boundary of the embryonic and extraembryonic tissue and the second repeller bisects the initial attractor, dividing the mesendoderm into regions that will give rise to the anterior and posterior primitive streak (Serra et al., 2020). This second repeller marks the position of the saddle bifurcation where the tissue flows diverge towards anterior and posterior (Cui et al., 2005; Fleury, 2012). At early elongation stages most of the tissue flows in the streak occurs from posterior to anterior; however, at later stages most of the flow is directed in the opposite direction, and the streak grows elongates backwards from this saddle point.

Ultimately, these flow organisers are the result of cell rearrangements, cell divisions and cell ingressions. The signature of these behaviours can be captured through the computation of the components of the strain rate from the velocity fields. The isotropic component of the strain rate reveals the detailed spatiotemporal patterns of tissue expansion and contraction as a result of cell divisions, ingressions and change in cell area; while the anisotropic component reveals the deformation produced by cell asymmetric shape changes and rearrangements (Fig. 2B).

### 3.2 The cell behaviours underlying the tissue flows

Many different cell behaviours have been proposed to underlie the formation of the PS from the cells located in the posterior sickle-shaped domain of mesendoderm precursors. These include directional cell divisions (Zahavi et al., 1998), directional cell intercalation (Lawson and Schoenwolf, 2001; Wei and Mikawa, 2000), chemotactic cell migration coupled with differential adhesion (Sandersius et al., 2011; Vasiev et al., 2010) and hypoblast anterior-posterior pulling of the epiblast (Fleury et al., 2015). Multiphoton *in-vivo* imaging of the nuclei of epiblast cells strongly suggested that the observed tissue flows were at least in part the resulting from cell intercalations during the formation of the PS (Voiculescu et al., 2007). The recent development of new transgenic lines of chick embryos in combination with in-vivo light-sheet microscopy now allows the detailed quantitative analysis of the spatiotemporal patterns of all critical cell behaviours, cell shape, cell ingression and cell intercalations that drive the formation of the PS and link them quantitatively with the large scale tissue flows and deformations (Rozbicki et al., 2015).

The formation of the PS is a biphasic process (Rozbicki et al., 2015). Shortly after the laying of the egg (HH1), mesendoderm cells located in a sickle-shaped domain in the posterior periphery of the AP start to intercalate on a large scale in a coordinated directional manner producing the convergence of the tissue towards the posterior midline. Later, the mesendoderm precursors extend along the midline of the embryo from posterior to anterior (HH2–3). At the same time, cells in the anterior epiblast rearrange to give way to the extending streak and move towards the lateral domains replacing the cells which previously moved to the PS (Chuai et al., 2006; Cui et al., 2005).

From the initial stages, many individual cell ingressions occur throughout the epiblast (Fig. 2C). The relevance of these scattered cell ingressions on the tissue mechanics is currently unclear but it could contribute to the fluidisation of the tissue (see below). However, later during the extension stages (HH2–3) cell ingressions become increasingly prominent in the streak, creating a tissue sink which contributes to pulling more cells (attractor) and at the same time counteracts the extension of the streak. Thanks to the widespread cell divisions on the surface of the embryo, the epiblast remains roughly constant in size even with massive cell ingressions occurring through the PS. Cell proliferation is fast, the cell cycle is around 6 h and uniform during the early stages of gastrulation and increases later in the boundary between the AO and AP later on (Cui et al., 2005). The analysis of the orientation of cell divisions has shown that initially during the early process of streak formation, the divisions in the mesendoderm are oriented perpendicular towards the anterior-posterior axis of the embryo, a readout of the tension built in the mesendoderm before gastrulation (Rozbicki et al., 2015).

### 3.3 Mechanisms driving cell intercalations

The directional intercalations in the mesendoderm are associated with extensive supracellular cables of phosphorylated myosin light chain spanning 2–8 cells (Fig. 3A). These cables are oriented in the direction of intercalation, perpendicular to the midline (Rozbicki et al., 2015). In the chick embryo, these cables form in a sickle-shaped domain mostly coincident with the mesendoderm and AO cells in the PMZ.

Cell intercalation has been thoroughly studied in amphibian mesoderm cells undergoing medio-lateral convergent extension (Keller, 2002). In this system, cells first polarise perpendicular to the axis of extension. The protrusive extensions of these bipolar polarised cells gain traction from neighbouring cells through cadherins, pulling them closer together in an actomyosin-dependent manner (Pfister et al., 2016; Skoglund et al., 2008). Chick mesendoderm cells have a strong epithelial character lacking very evident protrusions. There appear to be more similarities between cell intercalation in chick gastrulation and Drosophila germband extension (GBE) intercalation occurs through junction shrinking leading to T1 transitions and rosette formation driven by contraction of myosin cables in aligned junctions (Bertet et al., 2004; Fernandez-Gonzalez et al., 2009; Huebner and Wallingford, 2018).

In the chick embryo the inhibition of myosin I or myosin II activity blocks the formation of these cables, as well as, the intercalation, the apical contraction of the cells and the formation of the PS (Rozbicki et al., 2015). Furthermore, the local depolymerisation of the actin cytoskeleton, the substrate for the myosin motors, by the grafting of a Latrunculin-soaked bead in the base of the forming streak blocks the elongation of the tissue, while a Latrunculin-soaked bead grafted in the tip of the PS is carried along with the extending streak (Cui et al., 2005). These experiments suggest that intercalation of cells in the posterior streak are an essential part of the motor that drives the epithelial flows during streak extension. A recent report confirmed these findings of the myosin cables and provided model-prediction evidence that such a the transmission of forces along a contractile ring could drive the tissue flows in the AP assuming that it can be described as a viscous flow (Saadaoui et al., 2020).

While mesendoderm precursor cells in the posterior epiblast intercalate directionally, cells in the anterior epiblast cells initially rearrange in random directions, but ultimately they need to contribute to the tissue macroscopic flows (Fig. 3A). The initial absence of myosin cables outside the contractile sickle of mesendoderm precursors suggests the absence of longer-range coordination of intercalations. However, the cells in the anterior AP need to rearrange to accommodate the extending PS, without the bending or buckling of the epithelium, a process requiring a degree of tissue fluidity. Spontaneous fluctuations the junction lengths, due to stochastic variations in cortical myosin, are a possible source of randomly oriented cell intercalations contributing to tissue fluidisation in the Drosophila Notum (Curran et al., 2017).

Tissue fluidisation also can result from other cellular processes that change the local topology, such as cell divisions and cell ingressions (Fig. 3B). Blocking cell proliferation with aphidicolin (an inhibitor of the DNA polymerases δ and α) interferes with the generation of the distinctive rotational tissue flows and blocks the formation of the PS (Cui et al., 2005). This phenomenon has been attributed to the reduction of the epithelium fluidity due to the reduction of cell division mediated intercalation (Firmino et al., 2016), a process characterised by the separation of two daughter epithelial cells after division. Before cytokinesis, mitotic cells round-up close to the apical surface of the epiblast, greatly reducing the area of contact with neighbouring cells. These weakened interactions with their neighbours facilitate the intercalation of other cells between the two daughters after cytokinesis.

A reduction of the fluidity of the epiblast has also been reported to occur after treatment with hydroxyurea (an inhibitor of ribonucleotide reductase) and the apoptosis inhibitor QVDOPH, but surprisingly this treatment did not affect the tissue growth (Saadaoui et al., 2020). The coupling of cell division and cell intercalations is an attractive idea; however, the extent to which fluidity of the epithelium depends on cell divisions remains unclear. Mosaic inhibition of cell proliferation through the overexpression of the cyclin-kinase inhibitor p21/Waf severely limits the growth of the embryo but does not affect the formation of the PS (Chuai et al., 2006), which suggest that this level of inhibition has a great impact in the production of new cells without compromising the fluidity of the tissue.

Cell ingressions also have a significant impact on the topology of epithelial sheets which produce the rearrangement of the cells (Fig. 3B). During ingression, epithelial cells progressively reduce their apical area and perimeter to the point where they disappear from the surface. Depending on the mode of junction shortening the original neighbouring cells will all come into contact producing a multicellular rosette as described for GBE (Blankenship et al., 2006), the formation of zebrafish lateral line (Harding and Nechiporuk, 2012) and in the PS forming region in the chick embryo (Wagstaff et al., 2008). These rosettes are unstable structures whose resolution can only be asymmetrical, resulting in local tissue rearrangements and thereby contributing to tissue fluidity (Harding et al., 2014).

### 3.4 Signalling mechanisms that coordinate intercalations

The above processes explain which cell behaviours can result in intercalations, but do not yet explain how these intercalations are coordinated to drive the observed tissue flows. Intercalation of epithelial cells is a complex, active process requiring a remodelling of cell-cell interfaces in a precise spatiotemporal coordinated manner, known as T1 transitions. Since intercalation involves at least four cells, this requires the cytoskeletons of the intercalating cells to coordinate their activities and actively participate in the process. Identification of the signalling mechanisms that drive the formation of the oriented myosin cables in the posterior mesendoderm, responsible for the coordinated directional contraction and intercalation of the cells remains a critical mostly unresolved question.

The non-canonical WNT-dependent Planar Cell Polarity (PCP) signalling pathway has been implicated into the control of convergent-extension behaviour during gastrulation in amphibians (Ninomiya et al., 2004; Tada and Smith, 2000; Wallingford et al., 2000) and fish (Heisenberg et al., 2000). Interestingly the PCP signalling pathway was identified in Drosophila and found to be controlling polarised cell behaviours during the formation of bristle and hair patterns in Drosophila wing disks as well as and ommatidia in the eye, but it is not involved in controlling fly gastrulation (Humphries and Mlodzik, 2018).

In the chick embryo, the primary PCP ligand, *cWnt11*, is expressed at low levels throughout the epiblast and hypoblast before gastrulation (Skromne and Stern, 2001); however, the expression of a dominant-negative of *cWnt11* or a mutated version *Dishevelled* (a downstream mediator of the PCP pathway) was reported not to affect the tissue flows during primitive streak formation, but did affect the regression of the streak and extension of the embryo in later stages of development (Chuai et al., 2006). On the other hand in another study, the expression of Xenopus Dishevelled lacking the DEP domain produced a reduction of the convergence of the tissue and a partial block of streak formation in chick, as did the simultaneous knockdown of several PCP components expressed to varying degrees in the epiblast, *Celsr1* (*Flamingo1*), *Prickle1* and *Vangl2* (*Vangogh-like 2*) (Voiculescu et al., 2007). Furthermore, the rotation of the hypoblast which is known to result in the bending of the PS (Azar and Eyal-Giladi, 1981; Bertocchini and Stern, 2002; Foley et al., 2000; Voiculescu et al., 2007; Waddington and Gray, 1932) results in the ectopic expression of *Celsr1* and *Prickle1* in the epiblast, which could explain the influence of the hypoblast on streak formation (Voiculescu et al., 2007). The molecular mechanism linking expression of these PCP pathway components with the polarised activation of the acto-myosin cytoskeleton, which drives the directed cell intercalation, remains currently unknown in chick as well as other model organisms (Huebner and Wallingford, 2018; Huebner and Wallingford, 2018).

An alternative explanation is that the intercalations are oriented by a tension dependent self-organisation process (Fig. 3C). Detailed observations of cell behaviours during the onset of streak formation showed that the cell flows start close to the midline of the posterior sickle region followed by the onset of movement in more lateral regions (Rozbicki et al., 2015). The onset of motion spreads from medial to lateral, while cells flow from the periphery of the epiblast towards the midline. This implies the possible existence of a wave of mechanical tension spreading from the centre to the lateral regions of the mesendoderm, which polarises the cells and coordinates the directional intercalation at larger scales.

The simplest assumption would be that the propagation tension is mediated by the apical actomyosin cytoskeleton, including polarity complexes, adherens junctions and focal adhesion complexes (Gilmour et al., 2017; Lecuit et al., 2011). These complexes can detect and respond to mechanical perturbations providing the ingredients for a mechanically excitable medium (Bailles et al., 2019). Myosin II is directly and indirectly mechanosensitive (Diz-Muñoz et al., 2013; Iskratsch et al., 2014; Heissler and Sellers, 2016; Fernandez-Gonzalez et al., 2009); therefore, the contraction of a cell junction, due to the local activation of myosin II, followed by the subsequent increase of tension in neighbouring junctions, can result in the recruitment of myosin subsequently in those junctions and thus direct the formation of supracellular myosin cables in a tension dependent manner. This way, the dynamics of the myosin cables in the apical junctions could provide a readout of the junctional tension in the tissue. This has recently been confirmed through direct measurement of junctional tension via optical tweezing of individual junctions (Ferro et al., 2020). These experiments have shown that cells in the mesendoderm precursors in the posterior epiblast indeed show a higher tension than cells in the central region of epiblast and that a large tension component is Myosin dependent. Finally, cells ingress after reaching the streak, producing a local increase in tension which could be propagated laterally through the tension-dependent assembly of myosin cables. Since newcomers are continuously replacing the cells constituting the PS, this tension dependent mechanism of myosin cable formation could sustain the directional intercalation process of cells flowing towards to forming streak, contributing to its extension.

### 3.5 New insights into the mechanism of intercalation

In recent years, the molecular basis of epithelial cell intercalations has received notable attention. These studies lead to the identification of three other major players in the process of cell intercalation in addition to supracellular myosin cables, medial myosin and basal protrusions. These data mainly come from the study of the GBE in flies and their involvement in intercalation in other model systems remains to be established.

The picture emerging from Drosophila GBE is that cell intercalations associated with T1 transitions are driven by the combined action of junctional and medial myosin. Accumulation of myosin on a contracting junction gives rise to a fourfold vertex state, which is resolved by pulling out a new junction in a direction perpendicular to the direction of contraction requiring the action of medial myosin in the same two cells that initiated the contraction (Collinet et al., 2015). During GBE medial myosin drives pulsatile behaviour which drives oscillations in the apical area of the cells (Martin et al., 2009). These pulsations are linked to the shrinkage of junctions (Rauzi et al., 2010; Vanderleest et al., 2018), and the elongation of new ones (Bardet et al., 2013; Yu and Fernandez-Gonzalez, 2016). It remains to be seen whether this mechanism will apply to the observed T1 transitions in the chick embryo, once in-vivo monitoring of myosin dynamics becomes possible.

Epithelial cells are three-dimensional objects, and it is becoming evident that rearrangement of apical junctions is likely not sufficient to rearrange the cells. A combination of apical junction shrinking and basal crawling could work together to drive the intercalation (Huebner and Wallingford, 2018). Basal protrusion occurs in conjunction with junction shrinking during GBE (Sun et al., 2017). Basal protrusions are sometimes formed before the apical intercalation process starts and are independently regulated by Scr42/Rac1 signalling pathway (Sun et al., 2017). When the basal protrusions are abolished, GBE still occurs, but many rosette-mediated intercalations fail to resolve. Basal protrusions also play a major role in cell intercalations that drive convergent extension of neural epithelial cells in mouse embryos (Williams et al., 2014). Basal protrusions also occur in epiblast cells of chick embryos (Harrisson et al., 1991; Vakaet, 1984); however, a possible functional role in cell interactions still needs to be evaluated.

## 4 Internalisation of cells during gastrulation

During gastrulation, cell movements reorganise the single-layered embryo into a multi-layered tissue. This process is remarkably different among groups of vertebrates: While amphibians and fish create the inner layers by the movement of sheets of epithelial cells through the blastopore, birds and mammals do that by the ingression of individual cells, both in the epiblast and in the primitive streak. In the course of ingression, cells undergo an epithelial to mesenchymal transition (EMT), acquiring a motile phenotype, which allows them to migrate out of the streak to populate the mesoderm and the endoderm (Chuai et al., 2012; Yang et al., 2002).

In this section, we will summarise what is known about the spatial and temporal patterns of cell ingressions, the mechanisms of individual cell ingressions and signalling mechanisms that control them.

### 4.1 Sites of cell ingressions before and during streak formation

The first cell ingressions occur in early pre-gastrulation stages throughout the epiblast to form the hypoblast (Eyal-Giladi and Kochav, 1976; Kochav et al., 1980; Rozbicki et al., 2015; Voiculescu et al., 2014; Weinberger and Brick, 1982). Both the hypoblast and mesendoderm cells express the sugar epitope HNK-1, as well as randomly cells scattered in the epiblast (Canning and Stern, 1988). The targeted ablation of these cells abolishes streak formation; however, the formation of the body axis can be rescued by the grafting of mesoderm (Stern and Canning, 1990). This suggests that HNK1 expressing cells could ingress and form a population of pioneer cells which are necessary to initiate the process of streak formation (Voiculescu et al., 2014).

Recent investigations lead to the hypothesis that the massive cell ingressions in the streak are a form of collective behaviour (Voiculescu et al., 2014). In this model, ingressing cells secrete NODAL, which in turn induces other cells to ingress and release more NODAL, generating a positive feedback. NODAL also promotes the breakdown of the basement membrane (BM), one of the earliest signatures of ingression (Nakaya et al., 2008) and overexpression of LEFTY1, a NODAL inhibitor, blocks the accumulation of ingression rosettes in the posterior region of the embryo at early stages of streak formation (Yanagawa et al., 2011). This way, the concentration of NODAL-producing cells in the midline of the embryo due to the tissue flows in the epiblast could result in the formation of a collective ingression site in the streak (Voiculescu et al., 2014).

It remains unknown what triggers the ingression of these individual cells in the epiblast during the early stages of streak formation. Mechanical cues can inform cells about the homeostatic status of the epithelial layer, resulting in induction of cell divisions in tissues under tension (Gudipaty et al., 2017; Heisenberg, 2017), or non-apoptotic cell extrusions in overcrowded tissues (Eisenhoffer et al., 2012; Eisenhoffer and Rosenblatt, 2013). Similar mechanisms could be at play in avian gastrulation, the PS is likely an overcrowded region and subjected to high mechanical stress, possibly signalling cells to abandon the surface.

### 4.2 The mechanism of cell ingression

**Fig. 4:**
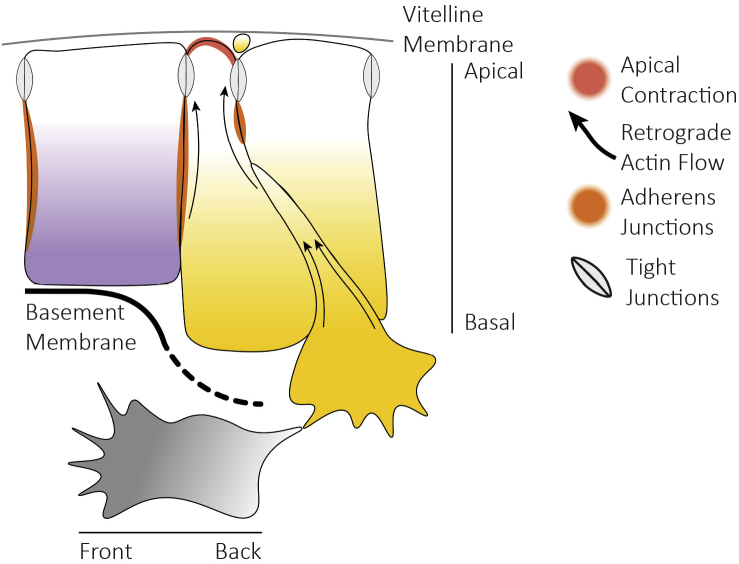
Mechanism of cell ingression. Before ingression, cells need to contract apically and disassemble tight and adherens junctions with their neighbours. During the process of ingression, cells get traction from the neighbour cells, extend protrusions and slide down, presumably, using a retrograde actin flow (black arrows). Finally, the cell acquires a mesenchymal phenotype and has converted the apicobasal polarity into a front-back polarity.

Cell ingression of individual cells starts with the contraction of the apical junctions, which pull neighbouring cells closer together, resulting in the formation of a rosette structure (Wagstaff et al., 2008). The cells then need to polarise, which involves the conversion of the apico-basal polarity in a front to back polarity, where the basal side of the ingressing cell becomes the leading edge of the migrating mesoderm cell and the apical side the trailing edge (Fig. 4).

During ingression, cells need to continuously remodel the tight and adherens junctions that are responsible for the epithelial cell-cell contacts to keep the epithelial integrity intact and finally dissolve them when exiting the epithelial layer. In order to generate the forces for the cells to move down, cells need to establish a retrograde actin flow from the leading edge of the cell towards the rear, which when coupled to cell-cell adhesion molecules can provide traction from neighbouring cells (Kametani and Takeichi, 2007). Therefore, an ingressing cell needs to undergo apical contraction, repolarise, activate its actomyosin cytoskeleton and dynamically tune its cell-cell contacts and cell-matrix contacts to exert and modulate traction. This mode of ingression resembles migration of an individual cell in a thin microfluidic channel, where get traction from the outside walls through a retrograde actin flow coupled to transmembrane adhesion molecules (Bergert et al., 2015). The difference is that during cell ingression, the cells get traction from the surrounding neighbours to dive into the embryo.

In the chick gastrulation, internalisation of cells through the streak requires a dramatic reshaping of the morphology of the cells. Ingressing cells contract apically acquiring a bottle shape and present basal protrusions which contact with the mesenchymal cells (Bancroft and Bellairs, 1974). Before ingression, epithelial cells show an increased basal blebbing associated with the degradation of the basement membrane (BM) (Harrisson et al., 1991; Nakaya et al., 2013; Vakaet, 1984).

There are few data on the mechanism and signals that control apical contraction and repolarisation of the actomyosin cytoskeleton in the chick embryo. Apical contraction is best understood in Drosophila; there, gastrulation starts with the apical contraction of cells in the mesendoderm precursors. These apical contractions are mediated by a reorganisation and activation of the apical actomyosin cytoskeleton. This involves the secretion of diffusible peptide ligand (short gastrulation, Fog) by the mesoderm cells. Binding of Fog to specific serpentine receptors results in the activation of a heterotrimeric G-protein (the G α subunit concertina) which turn on a Rho activator (Rho Gef2) that in turn, triggers Rho and Rho kinase, the ultimate regulators of actin-myosin localisation and activation (Dawes-Hoang et al., 2005; Jha et al., 2018; Kerridge et al., 2016; Manning et al., 2013). Except for the ligand, all other components of this signalling pathway are highly conserved in vertebrates. In Xenopus, it has been shown that Wnt5 might be the ligand needed for apical contraction and bottle cell formation (Choi and Sokol, 2009). There is some indication that inhibition of noncanonical WNT signalling through WNT5A/B and cWNT11 inhibits ingression of mesendoderm precursor cells in the streak of the chick embryo (Hardy et al., 2008). However, it remains to be determined whether and how this affects Rho signalling and actomyosin dynamics.

In many migrating cells, the polarisation of the leading edge and the rear of the cell involves the signal-dependent redistribution of phosphatidylinositol lipid signalling, with activation of PI3 kinase at the leading edge resulting in increased phosphatidyl inositide 3,4,5 trisphosphate (PI(3,4,5)P3) in the front of the cell and the phosphatidyl-3-phosphatase PTEN in the rear helping to maintain the gradient in PI(3,4,5)P3. In some cells an opposing gradient of PI(3,4)P2 defining the back has been shown to exit (Li et al., 2018). This phosphatidyl inositide signalling controls the activity of small GTPases such as RAC and CDC42 in the front, responsible actin proliferation in the leading edge, and RHO responsible for myosin activation and contraction in the back, reviewed in Saha et al. (2018). This configuration is maintained through a local positive feedback of actin polymerisation mediated by inositide phospholipids at the front, in combination with the long-range inhibition of actin polymerisation produced by the increase of membrane tension at the rear of the cell (Saha et al., 2018).

Inositide phospholipids are important markers of cellular architecture and epithelial polarity (Devreotes and Horwitz, 2015; Shewan et al., 2011). These signalling pathways are probably at play during cell ingressions; in fact, the overexpression of PTEN in the chick embryo blocks the process of EMT (Leslie et al., 2007), likely by interfering with the repolarisation of the epithelial cells into to front-back polarised mesenchymal cells during ingression.

### 4.3 Role of the basement membrane in polarity and signalling

Both in chick and mouse gastrulation, the degradation of the BM occurs before the onset of ingression in the streak (Nakaya et al., 2008; Williams et al., 2012). The inhibition of metalloproteases blocks streak formation and ingression (Mogi and Toyoizumi, 2010), suggesting that the breakdown of the BM is an essential process for ingression, which is likely mediated by the secretion of metalloproteases of the Adam family.

The digestion of the BM occurs after the destabilisation of the microtubules at the basal domain of the cells which, in turn, affects the interaction between the integrins and the BM (Nakaya et al., 2008). The interaction of the microtubules with the basal cells cortex seems to be mediated by CLIP-associated proteins (CLASPs), microtubule plus end interactors and the connection to the BM via transmembrane Dystroglycan (Nakaya et al., 2013). As expected, reduction of CLASP activity in epiblast cells results in degradation of the BM and premature EMT (Nakaya et al., 2013).The dismantling of the basal microtubule network correlates with the loss of RHOA and its activator NET1 in the basal domain, while overexpression of RHOA results in accumulation of cells in the streak and prevents the degradation of the BM (Nakaya et al., 2008). These experiments show that ingression is coupled with RHO inactivation at the basal side of the cell, which is likely part of the repolarisation process described above.

Furthermore, degradation of the BM likely affects integrin outside-in signalling and could affect the presence of critical signalling factors that interact with the basement membrane. Pharmacological inhibition of FGFRs also blocks the ingression through the PS, which may result from its effects on RHO activity since the expression of *RhoA* and *Net1* is dependent of FGF (Hardy et al., 2011).

### 4.4 Changes in adhesion

The separation of the mesendoderm and the ectoderm could in part be driven by differential cell adhesion. The differential RNA expression of the homophilic cadherins E-cadherin (Cdh1) and N-cadherin (Cdh2) between the epiblast and the mesendoderm, respectively, form the basis for this hypothesis (Hatta and Takeichi, 1986). In the cadherin-switch hypothesis, cells undergoing EMT repress the expression of E-cadherin and simultaneously activate N-cadherin expression. The repression of E-cadherin expression is controlled by SNAI2 (Snail2) (Cano et al., 2000), a transcription factor necessary for the EMT and a marker of mesendoderm cells (Nieto et al., 1994). This way, ingressing cells reduced the adhesion with the epiblast and enhanced the contacts with the inner layers.

Recently, the cadherin-switch model has been updated to show that the chick epiblast expresses P-cadherin as well as E-cadherin (Acloque et al., 2017; Moly et al., 2016). SNAI2 represses P-cadherin in the mesendoderm in the streak, while the transcription factor ZEB1 represses P-cadherin in the neural-plate (Acloque et al., 2017). Furthermore, only the ectoderm and the endoderm express the family of non-coding RNA miR-200 (Darnell et al., 2006), which blocks the translation of the transcription factors ZEB1 and ZEB2, two known repressors of E- and P-cadherins (Cano and Nieto, 2008).

However, the onset of ingression is not linked with the loss of E-cadherin or P-cadherin at the protein level (Hardy et al., 2011; Moly et al., 2016; Nakaya et al., 2008). Although ectopic expression of SNAI2 in the epiblast induced ingression of these cells in some studies (Acloque et al., 2011), several other studies failed to show ingression by the overexpression of SNAI2 (Hardy et al., 2011; Moly et al., 2016) and blocking of ingression by the inactivation of SNAI2 (Moly et al., 2016). It appears likely that ingression does not require reduction of P/E Cadherin, but requires the developmentally controlled expression of N-cadherin in mesendoderm precursor. It is currently unknown how N-cadherin expression is regulated at the transcriptional level, but it could be directly or indirectly under the control of SNAI2.

In addition to the downregulation of P-cadherin expression, SNAI2 could be essential for the determination of the embryonic territories. While SNAI2 is expressed only in the mesendoderm, the transcription factor *Sox3* is characteristic of the ectoderm (Rex et al., 1997). The reciprocal repression between SNAI2 and SOX3 define the mesendoderm and ectoderm territories, respectively (Acloque et al., 2011). Moreover, cells expressing SOX3 are unable to ingress through the PS. This way, a SNAI2-SOX3 gene regulatory network may contribute to a binary decision between epithelial and mesenchymal fates (Acloque et al., 2011).

### 4.5 Signalling mechanisms controlling ingression

The signals controlling the ingression of individual epiblast cells to form the hypoblast is yet largely unknown. A possibility would appear to be that it is controlled by mechanical cues involved in size control. Interestingly, in *Drosophila*, cell ingression is associated with quasi-periodic apical contraction cycles of an increasing number of cells (Martin et al., 2009). The coherence of these oscillations is dependent on the mesoderm transcription factor Snail, which can be replaced by a local mechanical indentation suggesting a Snail-dependent essential role for mechanics in the coordination of the ingression process (Pouille et al., 2009). Subsequently, it has been shown that tension does affect the internalisation cycle of the smog/fog receptors and also affect cadherin turnover (Jha et al., 2018; Kerridge et al., 2016). It remains to be determined whether these factors play a role in ingression in the chick embryo and what the connection is with WNT, FGF and TGFβ signalling, which all have been shown to directly or indirectly affect cell ingression.

## 5 Migration of mesendodermal cells to their targets

Once the cells have ingressed key remaining questions are, 1) What is the fate of the ingressing cells, which tissues and organs will they contribute to? 2) How do the cells know where to go and which signals guide them to their destination? 3) Which mechanisms control individual and collective cell movements and finally, 4) How do migrating cells affect the behaviour and differentiation of the surrounding cells?

These dynamic and reciprocal interactions between signalling and cell behaviour result in the emergent properties that control the morphogenesis at the tissue organ and organism level. They remain among the most challenging and fundamental questions in the study of development.

### 5.1 Fate of cells that ingress through the streak

The fate of mesodermal cells after their ingression through the streak has been the focus of many fate mapping studies. Initially, these were performed through DiI labelling of small groups of cells and more recently through expression of GFP constructs in scattered cells or in grafted cell populations (Hatada and Stern, 1994; Yang et al., 2008). The fate of the labelled cells was followed by either time-lapse cinematography or dissection and determination of the fate of the marked cells at various stages of development.

Cell fate mapping has shown that broadly speaking the first cells to ingress during extension of the streak are endoderm precursors and cells that will form the prechordal plate (Lawson and Schoenwolf, 2003). Later, when the streak is fully elongated, the node is formed, and the streak regresses as the cells from the node migrate to form the notochord and part of to the floor plate of the prospective neural tube (Gray and Dale, 2010; Selleck and Stern, 1991). The cells immediately behind the node give rise to the anterior somites, cells located in the middle streak from lateral plate mesoderm and the cells ingressing in the back of the streak form the extraembryonic mesoderm and the vasculature. Cells constituting the extraembryonic mesoderm will form the blood islands that will give rise to the extraembryonic circulatory system, as well as, structures in the embryonic circulatory system such as the heart (Chuai and Weijer, 2009a; Lawson and Schoenwolf, 2001; Psychoyos and Stern, 1996). With better labelling and imaging techniques, these maps will undoubtedly be further refined.

### 5.2 How much do the cells move individually?

Tracking individual mesendoderm cells shows that they cover very long distances in the embryo. How much do individual cells actively move to achieve this overall displacement and what constitutes the substrate that these cells move on? Do the cells use the extracellular matrix to get traction?

Antibody labelling of extracellular matrix components has shown that at least part of the extracellular matrix moves along with the cells, i.e. that the cells travel relatively little with respect to the matrix (Benazeraf et al., 2010; Czirok et al., 2004; Zamir et al., 2006). This would imply that the cells get little or no traction from the matrix and do not move actively over large distances. The question then remains which mechanisms transport or deform the matrix over large distances. A possibility is that individual mesendoderm cells from a cellular scaffold via specific cell-cell contacts from which other cells get traction and transport the matrix along with them. In this mechanism, the movement of the cells within this meshwork determines the overall cell flow, analogous to epithelial tissues where the intercalation of many cells results in large macroscopic displacements of cell positions with respect to an external observer (Chuai and Weijer, 2009b).

### 5.3 Signals that direct cells to the correct place

How are cells directed towards the correct position in the embryo? Simple swapping experiments showed that the cells are not pre-programmed to go to a particular place (Yang et al., 2002). Middle streak cells grafted in the anterior streak will form somites, instead of lateral plate mesoderm and reversely, anterior streak cells placed in the middle of the streak will form lateral plate mesoderm. This suggests that mesendoderm express or can rapidly induce expression of a variety of receptors and that their fate depends on the signals that they encounter. Growth factors of the FGF, VEGF, PDGF families have all been implicated in the control of mesendoderm cell migration in the chick embryo.

**Fig. 5:**
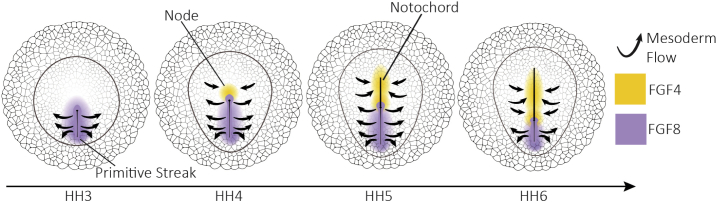
FGF signalling in mesoderm migration. 1) FGF8 is secreted in the primitive streak and act as a repulsive signal for the mesoderm cells. Once cells ingress they migrate out via chemo-repulsion. 2) When the node starts to regress it creates the notochord which secretes FGF4 and attracts migrant mesoderm cells back towards the midline.

FGF receptors are expressed in the ectoderm and migrating mesoderm cells (Hardy et al., 2011; Lunn et al., 2007). FGF4 is expressed in the node, the forming notochord and the streak, and FGF8 is strongly expressed in the streak and migrating mesoderm cells (Shamim and Mason, 1999; Stolte et al., 2002). FGF4 and FGF8 have been shown to as attractant and repellent for streak cells, respectively (Yang et al., 2002). Based on the expression patterns of FGF4 and FGF8, it has been proposed that after ingression, the cells initially move away from the streak in response to the repulsive action of FGF8. When the node starts to regress, and the notochord starts to form FGF4 signals from the forming notochord attract the cells back in towards the midline where they form somites. The mesendoderm cells more posterior in the streak migrate for longer outward before they perceive the attracting signals from the forming notochord resulting in them making longer excursions and taking up more lateral positions (Chuai and Weijer, 2009a; Yang et al., 2002). This model could explain how the anterior-posterior position of cells in the streak is translated into a mediolateral position in the later stages of development (Fig. 5).

Based on the directionality of the cell trajectories of mesendoderm precursor cells in the embryo and their directional movement away from ectopic FGF8 signals, it was proposed that the cells moved by chemo repulsion (Yang et al., 2002). However, it has been suggested that FGF8 could act as a chemokinetic factor for the movement of the presomitic mesoderm later in the regression stages (Benazeraf et al., 2010). This is based on the observation that the movement of cells relative to the extracellular matrix appears random as discussed above and that this relative motility is modulated by FGF8, higher concentrations resulting in faster movement. This way, the expression of FGF in the streak could result in a biased random walk away from the streak. Investigation of the detailed motility mechanism will require further characterisation of the cell behaviours under better defined conditions in-vitro.

Posterior streak cells express the KDR receptor (VEGFR2) (Eichmann et al., 1997), show a directed chemotactic response to various VEGFA, C and D isoforms and migrate out of the streak in response to VEGF signals (Chuai et al., 2012; Eichmann et al., 1998). VEGF signalling is complex, a variety of VEGF's are expressed in the gastrulation stage chick embryo, but their precise spatiotemporal localisation remains to be determined.

Another factor belonging to the split kinase domain family of growth factor receptors often implicated in the control of cell movement during development is the PDGF receptor. PDGF signalling has been implicated in directing the collective movement of leading-edge mesoderm cells in the Xenopus embryo through suppressing contact inhibition of movement (Nagel and Winklbauer, 2018). In the chick embryo, PDGFA is expressed in the epiblast while the PDGFRA receptor is found in the migrating mesoderm cells (Yang et al., 2008). Downregulation of PDGF results in an inhibition of cell movement, but the ectopic expression of PDGF failed to show clear evidence for a chemotactic response. PDGF signalling is required for functional N-cadherin expression, through regulation at the posttranslational level, suggesting that PDGF signalling facilitates the collective movement of mesoderm cells through the control of the expression of N-cadherin on migrating cells (Yang et al., 2008).

Ectopic application of WNTs and BMP's has been shown to affect the characteristic migration patterns of cardiac precursor cells (Sweetman et al., 2008; Yue et al., 2008). In the case of WNT3A and BMP2, the effects of both factors are mediated via SMAD1 signalling, suggesting that these factors affect transcriptional programs that impair the ability of the cells to interpret guidance signals, but the molecular details remain to be resolved. Wnt3A signalling has also been shown to act upstream of RHOA activation, suggesting an effect on cell polarisation (Song et al., 2014).

It remains to be determined whether there is a role for other small molecule attractants that act through serpentine receptor G protein-coupled signalling pathways directing the migration of mesoderm and endoderm cells in the chick embryo. A relatively well-characterised chemokine that controls cell migration in early development is SDF1, which signals through the CXCR4 and CXCR7 receptors to control the migration of primordial germ cells in zebrafish and mouse, as well as, the migration of endoderm cells in zebrafish (Knaut et al., 2003; Mizoguchi et al., 2008; Molyneaux et al., 2003). These factors and receptors are all expressed in avian embryos, but their role in the control of primordial germ cell migration remains to be elucidated (Stebler et al., 2004). Another exciting family of small peptides such as apelin and Toddler act through the G protein-coupled apelin receptor which has also been shown to affect migration of mesoderm cells in zebrafish embryos (Pauli et al., 2014; Zeng et al., 2007).

The identification and analysis of the *in-vivo* guidance signals that direct the mesendoderm cells to their correct location as well as the mechanisms of migration of the mesendoderm cells in the gastrulating chick embryo are critical to understanding gastrulation. Methods to visualise the cell behaviours and modes of migration of the mesendoderm cells in the context of the embryo are rapidly improving. The emergence of vastly improved transgenesis techniques will help in the mutational analysis of signals and cellular responses. A very challenging problem remains the measurement of the spatiotemporal organisation of the signalling dynamics during development. This will be crucial for a mechanistic understanding of the modes and regulation of mesendoderm cell migration, as well as the effects that the migrating cells exert on surrounding tissues.

## 6 Emerging technical advances

Quantitative analysis of the dynamic interactions between signalling and cell movement behaviours is essential to the mechanistic understanding of the emergent properties that drive tissue formation and morphogenesis during gastrulation. The chick embryo is expected to continue to contribute greatly to our understanding of amniote gastrulation. Existing methods allow extensive mechanical and chemical perturbation in combination with gene knockdown and overexpression techniques. This, together with the rapid development in large scale light-sheet microscopy live imaging capabilities and computational methodology required for the large scale data analysis are expected to advance these investigations to a new level.

A drawback of the avian system has been the relatively long timespans required for existing transgenesis methods to generate stable genetic modifications required for targeted labelling and expression of specific gene products and mutational analysis. This is, however, changing rapidly, breakthrough developments are the ability to cultivate primordial germ cells, which give access to manipulation of the genome using well established cell culture manipulation techniques, such CRISPR-Cas9 mediated gene knock-in and knock-out techniques (Idoko-Akoh et al., 2018). The primordial germ cells can then be injected in early chick embryos, where they will directly target the germline. When this is done in sterile surrogate hosts, this will enable the rapid establishment of novel transgenic lines, without the need for extensive crossing (Woodcock et al., 2019). In quail embryos transgenesis can be accomplished by transfecting circulating primordial germ cells *in vivo* (Serralbo et al., 2020). These developments in genetic tractability and manipulation will undoubtedly greatly further enhance the importance of the avian embryo as a model system for morphogenesis.

## References

[bb0005] Acloque H., Ocana O.H., Matheu A., Rizzoti K., Wise C., Lovell-Badge R., Nieto M.A. (2011). Reciprocal repression between Sox3 and Snail transcription factors defines embryonic territories at gastrulation. Dev. Cell.

[bb0010] Acloque H., Ocana O.H., Abad D., Stern C.D., Nieto M.A. (2017). Snail2 and Zeb2 repress P-cadherin to define embryonic territories in the chick embryo. Development.

[bb0015] Alev C., Wu Y.P., Nakaya Y., Sheng G.J. (2013). Decoupling of amniote gastrulation and streak formation reveals a morphogenetic unity in vertebrate mesoderm induction. Development.

[bb0020] Arendt D., Nubler-Jung K. (1999). Rearranging gastrulation in the name of yolk: evolution of gastrulation in yolk-rich amniote eggs. Mech. Dev..

[bb0025] Arias C.F., Herrero M.A., Stern C.D., Bertocchini F. (2017). A molecular mechanism of symmetry breaking in the early chick embryo. Sci. Rep..

[bb0030] Azar Y., Eyal-Giladi H. (1979). Marginal zone cells—the primitive streak-inducing component of the primary hypoblast in the chick. Development.

[bb0035] Azar Y., Eyal-Giladi H. (1981). Interaction of epiblast and hypoblast in the formation of the primitive streak and the embryonic axis in chick, as revealed by hypoblast-rotation experiments. Development.

[bb0040] Bailles A., Collinet C., Philippe J.M., Lenne P.F., Munro E., Lecuit T. (2019). Genetic induction and mechanochemical propagation of a morphogenetic wave. Nature.

[bb0045] Bancroft M., Bellairs R. (1974). The onset of differentiation in the epiblast of the chick blastoderm (SEM and TEM). Cell Tissue Res..

[bb0050] Bardet P.L., Guirao B., Paoletti C., Serman F., Léopold V., Bosveld F., Goya Y., Mirouse V., Graner F., Bellaïche Y. (2013). PTEN controls junction lengthening and stability during cell rearrangement in epithelial tissue. Dev. Cell.

[bb0055] Beccari L., Moris N., Girgin M., Turner D.A., Baillie-Johnson P., Cossy A.C., Lutolf M.P., Duboule D., Arias A.M. (2018). Multi-axial self-organization properties of mouse embryonic stem cells into gastruloids. Nature.

[bb0060] Bellairs R., Boyde A., Heaysman J. (1969). The relationship between the edge of the chick blastoderm and the vitelline membrane. Wilhelm Roux'Archiv fur Entwicklungsmechanik der Organismen.

[bb0065] Benazeraf B., Francois P., Baker R.E., Denans N., Little C.D., Pourquie O. (2010). A random cell motility gradient downstream of FGF controls elongation of an amniote embryo. Nature.

[bb0070] Bergert M., Erzberger A., Desai R.A., Aspalter I.M., Oates A.C., Charras G., Salbreux G., Paluch E.K. (2015). Force transmission during adhesion-independent migration. Nat. Cell Biol..

[bb0075] Bertet C., Sulak L., Lecuit T. (2004). Myosin-dependent junction remodelling controls planar cell intercalation and axis elongation. Nature.

[bb0080] Bertocchini F., Stern C.D. (2002). The hypoblast of the chick embryo positions the primitive streak by antagonizing nodal signaling. Dev. Cell.

[bb0085] Bertocchini F., Stern C.D. (2012). Gata2 provides an early anterior bias and uncovers a global positioning system for polarity in the amniote embryo. Development.

[bb0090] Bertocchini F., Skromne I., Wolpert L., Stern C.D. (2004). Determination of embryonic polarity in a regulative system: evidence for endogenous inhibitors acting sequentially during primitive streak formation in the chick embryo. Development.

[bb0095] Blankenship J.T., Backovic S.T., Sanny J.S., Weitz O., Zallen J.A. (2006). Multicellular rosette formation links planar cell polarity to tissue morphogenesis. Dev. Cell.

[bb0100] Callebaut M. (2005). Origin, fate, and function of the components of the avian germ disc region and early blastoderm: role of ooplasmic determinants. Developmental dynamics: an official publication of the American Association of Anatomists.

[bb0105] Callebaut M., Van Nueten E. (1994). Rauber's (Koller's) sickle: the early gastrulation organizer of the avian blastoderm. Eur. J. Morphol..

[bb0110] Callebaut M., Van Nueten E., Harrisson F., Bortier H. (2005). Early interaction between deep and superficial layers in avian blastodiscs: uptake of ooplasmic determinants. Belgian journal of zoology.

[bb0115] Canning D.R., Stern C.D. (1988). Changes in the expression of the carbohydrate epitope HNK-1 associated with mesoderm induction in the chick embryo. Development.

[bb0120] Cano A., Nieto M.A. (2008). Non-coding RNAs take centre stage in epithelial-to-mesenchymal transition. Trends Cell Biol..

[bb0125] Cano A., Pérez-Moreno M.A., Rodrigo I., Locascio A., Blanco M.J., del Barrio M.G., Portillo F., Nieto M.A. (2000). The transcription factor snail controls epithelial-mesenchymal transitions by repressing E-cadherin expression. Nat. Cell Biol..

[bb0130] Choi S.-C., Sokol S.Y. (2009). The involvement of lethal giant larvae and Wnt signaling in bottle cell formation in Xenopus embryos. Dev. Biol..

[bb0135] Chuai M., Weijer C.J. (2009). Regulation of cell migration during chick gastrulation. Curr. Opin. Genet. Dev..

[bb0140] Chuai M., Weijer C.J. (2009). Who moves whom during primitive streak formation in the chick embryo. HFSP J.

[bb0145] Chuai M., Zeng W., Yang X., Boychenko V., Glazier J.A., Weijer C.J. (2006). Cell movement during chick primitive streak formation. Dev. Biol..

[bb0150] Chuai M., Hughes D., Weijer C.J. (2012). Collective epithelial and mesenchymal cell migration during gastrulation. Curr Genomics.

[bb0155] Clavert J., Abercrombie M., Brachet J. (1962). Symmetrization of the egg of vertebrates. Advances in Morphogenesis.

[bb0160] Collinet C., Rauzi M., Lenne P.-F., Lecuit T. (2015). Local and tissue-scale forces drive oriented junction growth during tissue extension. Nat. Cell Biol..

[bb0165] Cui C., Yang X., Chuai M., Glazier J.A., Weijer C.J. (2005). Analysis of tissue flow patterns during primitive streak formation in the chick embryo. Dev. Biol..

[bb0170] Curran S., Strandkvist C., Bathmann J., de Gennes M., Kabla A., Salbreux G., Baum B. (2017). Myosin II controls junction fluctuations to guide epithelial tissue ordering. Dev. Cell.

[bb0175] Czirok A., Rongish B.J., Little C.D. (2004). Extracellular matrix dynamics during vertebrate axis formation. Dev. Biol..

[bb0180] Darnell D.K., Kaur S., Stanislaw S., Konieczka J.H., Konieczka J.K., Yatskievych T.A., Antin P.B. (2006). MicroRNA expression during chick embryo development. Dev. Dyn..

[bb0185] Dawes-Hoang R.E., Parmar K.M., Christiansen A.E., Phelps C.B., Brand A.H., Wieschaus E.F. (2005). Folded gastrulation, cell shape change and the control of myosin localization. Development.

[bb0190] Devreotes P., Horwitz A.R. (2015). Signaling networks that regulate cell migration. Cold Spring Harb. Perspect. Biol..

[bb0195] Diz-Muñoz A., Fletcher D.A., Weiner O.D. (2013). Use the force: membrane tension as an organizer of cell shape and motility. Trends Cell Biol..

[bb0200] Downie J.R. (1976). The mechanism of chick blastoderm expansion. J Embryol Exp Morphol.

[bb0205] Eichmann A., Corbel C., Nataf V., Vaigot P., Breant C., Le Douarin N.M. (1997). Ligand-dependent development of the endothelial and hemopoietic lineages from embryonic mesodermal cells expressing vascular endothelial growth factor receptor 2. Proc. Natl. Acad. Sci. U. S. A..

[bb0210] Eichmann A., Corbel C., Jaffredo T., Breant C., Joukov V., Kumar V., Alitalo K., le Douarin N.M. (1998). Avian VEGF-C: cloning, embryonic expression pattern and stimulation of the differentiation of VEGFR2-expressing endothelial cell precursors. Development.

[bb0215] Eisenhoffer G.T., Rosenblatt J. (2013). Bringing balance by force: live cell extrusion controls epithelial cell numbers. Trends Cell Biol..

[bb0220] Eisenhoffer G.T., Loftus P.D., Yoshigi M., Otsuna H., Chien C.B., Morcos P.A., Rosenblatt J. (2012). Crowding induces live cell extrusion to maintain homeostatic cell numbers in epithelia. Nature.

[bb0225] Eyal-Giladi H., Fabian B.C. (1980). Axis determination in uterine chick blastodiscs under changing spatial positions during the sensitive period for polarity. Dev. Biol..

[bb0230] Eyal-Giladi H., Khaner O. (1989). The chick's marginal zone and primitive streak formation: II. Quantification of the marginal zone's potencies—temporal and spatial aspects. Dev. Biol..

[bb0235] Eyal-Giladi H., Kochav S. (1976). From cleavage to primitive streak formation: a complementary normal table and a new look at the first stages of the development of the chick. I. General morphology. Dev. Biol..

[bb0240] Fernandez-Gonzalez R., Simoes S.e.M., Röper J.C., Eaton S., Zallen J.A. (2009). Myosin II dynamics are regulated by tension in intercalating cells. Dev. Cell.

[bb0245] Ferro V., Chuai M., McGloin D., Weijer C.J. (2020). Measurement of junctional tension in epithelial cells at the onset of primitive streak formation in the chick embryo via non-destructive optical manipulation. Development.

[bb0250] Firmino J., Rocancourt D., Saadaoui M., Moreau C., Gros J. (2016). Cell division drives epithelial cell rearrangements during gastrulation in chick. Dev. Cell.

[bb5005] Fleury V. (2012). Clarifying tetrapod embryogenesis by a dorso-ventral analysis of the tissue flows during early stages of chicken development. Bio Systems.

[bb0255] Fleury V., Chevalier N.R., Furfaro F., Duband J.L. (2015). Buckling along boundaries of elastic contrast as a mechanism for early vertebrate morphogenesis. European Physical Journal E.

[bb0260] Foley A.C., Skromne I., Stern C.D. (2000). Reconciling different models of forebrain induction and patterning: a dual role for the hypoblast. Development.

[bb0265] Gilmour D., Rembold M., Leptin M. (2017). From morphogen to morphogenesis and back. Nature.

[bb0270] Gräper L. (1929). Die Primitiventwicklung des Hühnchens nach stereokinematographischen Untersuchungen, kontrolliert durch vitale Farbmarkierung und verglichen mit der Entwicklung anderer Wirbeltiere. Dev. Genes Evol..

[bb5010] Gray S.D., Dale J.K. (2010). Notch signalling regulates the contribution of progenitor cells from the chick Hensen's node to the floor plate and notochord. Development.

[bb0275] Gudipaty S.A., Lindblom J., Loftus P.D., Redd M.J., Edes K., Davey C.F., Krishnegowda V., Rosenblatt J. (2017). Mechanical stretch triggers rapid epithelial cell division through Piezo1. Nature.

[bb0280] Harding M.J., Nechiporuk A.V. (2012). Fgfr-Ras-MAPK signaling is required for apical constriction via apical positioning of Rho-associated kinase during mechanosensory organ formation. Development.

[bb0285] Harding M.J., McGraw H.F., Nechiporuk A. (2014). The roles and regulation of multicellular rosette structures during morphogenesis. Development.

[bb0290] Hardy K.M., Garriock R.J., Yatskievych T.A., D'Agostino S.L., Antin P.B., Krieg P.A. (2008). Non-canonical Wnt signaling through Wnt5a/b and a novel Wnt11 gene, Wnt11b, regulates cell migration during avian gastrulation. Dev. Biol..

[bb0295] Hardy K.M., Yatskievych T.A., Konieczka J.H., Bobbs A.S., Antin P.B. (2011). FGF signalling through RAS/MAPK and PI3K pathways regulates cell movement and gene expression in the chicken primitive streak without affecting E-cadherin expression. BMC Dev. Biol..

[bb0300] Harrisson F., Callebaut M., Vakaet L. (1991). Features of polyingression and primitive streak ingression through the basal lamina in the chicken blastoderm. Anat. Rec..

[bb0305] Hatada Y., Stern C.D. (1994). A fate map of the epiblast of the early chick-embryo. Development.

[bb0310] Hatta K., Takeichi M. (1986). Expression of N-cadherin adhesion molecules associated with early morphogenetic events in chick development. Nature.

[bb0315] Heisenberg C.P. (2017). Cell biology stretched divisions. Nature.

[bb0320] Heisenberg C.P., Tada M., Rauch G.J., Saúde L., Concha M.L., Geisler R., Stemple D.L., Smith J.C., Wilson S.W. (2000). Silberblick/Wnt11 mediates convergent extension movements during zebrafish gastrulation. Nature.

[bb0325] Heissler S.M., Sellers J.R. (2016). Kinetic adaptations of myosins for their diverse cellular functions. Traffic.

[bb0330] Huebner R.J., Wallingford J.B. (2018). Coming to consensus: a unifying model emerges for convergent extension. Dev. Cell.

[bb0335] Hume C.R., Dodd J. (1993). Cwnt-8C: a novel Wnt gene with a potential role in primitive streak formation and hindbrain organization. Development.

[bb0340] Humphries A.C., Mlodzik M. (2018). From instruction to output: Wnt/PCP signaling in development and cancer. Curr. Opin. Cell Biol..

[bb0345] Idoko-Akoh A., Taylor L., Sang H.M., McGrew M.J. (2018). High fidelity CRISPR/Cas9 increases precise monoallelic and biallelic editing events in primordial germ cells. Sci. Rep..

[bb0350] Iskratsch T., Wolfenson H., Sheetz M.P. (2014). Appreciating force and shape—the rise of mechanotransduction in cell biology. Nat Rev Mol Cell Biol.

[bb0355] Jha A., van Zanten T.S., Philippe J.M., Mayor S., Lecuit T. (2018). Quantitative control of GPCR organization and signaling by endocytosis in epithelial morphogenesis. Curr. Biol..

[bb0360] Kametani Y., Takeichi M. (2007). Basal-to-apical cadherin flow at cell junctions. Nat. Cell Biol..

[bb0365] Keller R. (2002). Shaping the vertebrate body plan by polarized embryonic cell movements. Science.

[bb0370] Kerridge S., Munjal A., Philippe J.-M., Jha A., de las Bayonas A.G., Saurin Andrew J., Lecuit T. (2016). Modular activation of Rho1 by GPCR signalling imparts polarized myosin II activation during morphogenesis. Nat. Cell Biol..

[bb0375] Khaner O., Eyalgiladi H. (1989). The chicks marginal zone and primitive streak formation. 1. Coordinative effect of induction and inhibition. Dev. Biol..

[bb0380] Knaut H., Werz C., Geisler R., Nusslein-Volhard C. (2003). A zebrafish homologue of the chemokine receptor Cxcr4 is a germ-cell guidance receptor. Nature.

[bb0385] Kochav S., Eyal-Giladi H. (1971). Bilateral symmetry in chick embryo determination by gravity. Science.

[bb0390] Kochav S., Ginsburg M., Eyal-Giladi H. (1980). From cleavage to primitive streak formation: a complementary normal table and a new look at the first stages of the development of the chick: II. Microscopic anatomy and cell population dynamics. Dev. Biol..

[bb0395] Lawson A., Schoenwolf G.C. (2001). Cell populations and morphogenetic movements underlying formation of the avian primitive streak and organizer. Genesis.

[bb0400] Lawson A., Schoenwolf G.C. (2003). Epiblast and primitive-streak origins of the endoderm in the gastrulating chick embryo. Development.

[bb0405] Lecuit T., Lenne P.-F., Munro E. (2011). Force generation, transmission, and integration during cell and tissue morphogenesis. Annu. Rev. Cell Dev. Biol..

[bb0410] Lee H.C., Lu H.-C., Turmaine M., Oliveira N.M., Yang Y., De Almeida I., Stern C.D. (2020). Molecular anatomy of the pre-primitive-streak chick embryo. Open Biol..

[bb0415] Leslie N.R., Yang X., Downes C.P., Weijer C.J. (2007). PtdIns (3, 4, 5) P3-dependent and-independent roles for PTEN in the control of cell migration. Curr. Biol..

[bb0420] Li X., Edwards M., Swaney K.F., Singh N., Bhattacharya S., Borleis J., Long Y., Iglesias P.A., Chen J., Devreotes P.N. (2018). Mutually inhibitory Ras-PI(3,4)P_2_ feedback loops mediate cell migration. Proc. Natl. Acad. Sci..

[bb0425] Lunn J.S., Fishwick K.J., Halley P.A., Storey K.G. (2007). A spatial and temporal map of FGF/Erk1/2 activity and response repertoires in the early chick embryo. Dev. Biol..

[bb0430] Lutz H. (1949). Sur le production experimentale de la polyembryonie et de la monstruosite double chez les oiseaux. Arch. Anat. Microsc. Morph.Exp..

[bb0435] Manning A.J., Peters K.A., Peifer M., Rogers S.L. (2013). Regulation of epithelial morphogenesis by the g protein-coupled receptor mist and its ligand fog. Sci. Signal..

[bb0440] Martin A.C., Kaschube M., Wieschaus E.F. (2009). Pulsed contractions of an actin–myosin network drive apical constriction. Nature.

[bb0445] Mizoguchi T., Verkade H., Heath J.K., Kuroiwa A., Kikuchi Y. (2008). Sdf1/Cxcr4 signaling controls the dorsal migration of endodermal cells during zebrafish gastrulation. Development.

[bb0450] Mogi K., Toyoizumi R. (2010). Invasion by matrix metalloproteinase-expressing cells is important for primitive streak formation in early chick blastoderm. Cells Tissues Organs.

[bb0455] Moly P.K., Cooley J.R., Zeltzer S.L., Yatskievych T.A., Antin P.B. (2016). Gastrulation EMT is independent of P-cadherin downregulation. PLoS One.

[bb0460] Molyneaux K.A., Zinszner H., Kunwar P.S., Schaible K., Stebler J., Sunshine M.J., O'Brien W., Raz E., Littman D., Wylie C., Lehmann R. (2003). The chemokine SDF1/CXCL12 and its receptor CXCR4 regulate mouse germ cell migration and survival. Development.

[bb0465] Nagel M., Winklbauer R. (2018). PDGF-A suppresses contact inhibition during directional collective cell migration. Development.

[bb0470] Nakaya Y., Sukowati E.W., Wu Y., Sheng G.J. (2008). RhoA and microtubule dynamics control cell-basement membrane interaction in EMT during gastrulation. Nat. Cell Biol..

[bb0475] Nakaya Y., Sukowati E.W., Sheng G. (2013). Epiblast integrity requires CLASP and Dystroglycan-mediated microtubule anchoring to the basal cortex. J. Cell Biol..

[bb0480] Nieto M.A., Sargent M.G., Wilkinson D.G., Cooke J. (1994). Control of cell behavior during vertebrate development by Slug, a zinc finger gene. Science.

[bb0485] Ninomiya H., Elinson R.P., Winklbauer R. (2004). Antero-posterior tissue polarity links mesoderm convergent extension to axial patterning. Nature.

[bb0490] Pasteels J. (1940). Un apercu comparatif de la gastrulation chez les chordes. Biol. Rev..

[bb0495] Pauli A., Norris M.L., Valen E., Chew G.-L., Gagnon J.A., Zimmerman S., Mitchell A., Ma J., Dubrulle J., Reyon D., Tsai S.Q., Joung J.K., Saghatelian A., Schier A.F. (2014). Toddler: an embryonic signal that promotes cell movement via apelin receptors. Science.

[bb0500] Perea-Gomez A., Camus A., Moreau A., Grieve K., Moneron G., Dubois A., Cibert C., Collignon J. (2004). Initiation of gastrulation in the mouse embryo is preceded by an apparent shift in the orientation of the anterior-posterior axis. Curr. Biol..

[bb0505] Pfister K., Shook D.R., Chang C., Keller R., Skoglund P. (2016). Molecular model for force production and transmission during vertebrate gastrulation. Development.

[bb0510] Pouille P.A., Ahmadi P., Brunet A.C., Farge E. (2009). Mechanical signals trigger Myosin II redistribution and mesoderm invagination in Drosophila embryos. Sci. Signal..

[bb0515] Psychoyos D., Stern C.D. (1996). Fates and migratory routes of primitive streak cells in the chick embryo. Development.

[bb0520] Raffaelli A., Stern C.D. (2019). Signaling events regulating embryonic polarity and formation of the primitive streak in the chick embryo. Gastrulation: From Embryonic Pattern to Form.

[bb0525] Rauzi M., Lenne P.-F., Lecuit T. (2010). Planar polarized actomyosin contractile flows control epithelial junction remodelling. Nature.

[bb0530] Rex M., Orme A., Uwanogho D., Tointon K., Wigmore P.M., Sharpe P.T., Scotting P.J. (1997). Dynamic expression of chicken Sox2 and Sox3 genes in ectoderm induced to form neural tissue. Dev. Dyn..

[bb0535] Rozbicki E., Chuai M., Karjalainen A.I., Song F., Sang H.M., Martin R., Knolker H.J., MacDonald M.P., Weijer C.J. (2015). Myosin-II-mediated cell shape changes and cell intercalation contribute to primitive streak formation. Nat. Cell Biol..

[bb0540] Saadaoui M., Rocancourt D., Roussel J., Corson F., Gros J. (2020). A tensile ring drives tissue flows to shape the gastrulating amniote embryo. Science.

[bb0545] Saha S., Nagy T.L., Weiner O.D. (2018). Joining forces: crosstalk between biochemical signalling and physical forces orchestrates cellular polarity and dynamics. Philosophical Transactions of the Royal Society B: Biological Sciences.

[bb0550] Sandersius S.A., Chuai M., Weijer C.J., Newman T.J. (2011). A ‘chemotactic dipole’ mechanism for large-scale vortex motion during primitive streak formation in the chick embryo. Phys. Biol..

[bb0555] Seleiro E.A., Connolly D.J., Cooke J. (1996). Early developmental expression and experimental axis determination by the chicken Vg1 gene. Current biology : CB.

[bb0560] Selleck M.A., Stern C.D. (1991). Fate mapping and cell lineage analysis of Hensen's node in the chick embryo. Development.

[bb0565] Serra M., Streichan S., Chuai M., Weijer C.J., Mahadevan L. (2020). Dynamic morphoskeletons in development. Proc. Natl. Acad. Sci..

[bb5020] Serralbo O., Salgado D., Véron N., Cooper C., Dejardin M.-J., Doran T., Gros J., Marcelle C. (2020). Transgenesis and web resources in quail. Elife.

[bb0570] Shah S.B., Skromne I., Hume C.R., Kessler D.S., Lee K.J., Stern C.D., Dodd J. (1997). Misexpression of chick Vg1 in the marginal zone induces primitive streak formation. Development.

[bb0575] Shamim H., Mason I. (1999). Expression of Fgf4 during early development of the chick embryo. Mech. Dev..

[bb0580] Sheng G. (2014). Day-1 chick development. Developmental dynamics: an official publication of the American Association of Anatomists.

[bb0585] Shewan A., Eastburn D.J., Mostov K. (2011). Phosphoinositides in cell architecture. Cold Spring Harb. Perspect. Biol..

[bb0590] Simunovic M., Metzger J.J., Etoc F., Yoney A., Ruzo A., Martyn I., Croft G., You D.S., Brivanlou A.H., Siggia E.D. (2019). A 3D model of a human epiblast reveals BMP4-driven symmetry breaking. Nat. Cell Biol..

[bb0595] Skoglund P., Rolo A., Chen X., Gumbiner B.M., Keller R. (2008). Convergence and extension at gastrulation require a myosin IIB-dependent cortical actin network. Development.

[bb0600] Skromne I., Stern C.D. (2001). Interactions between Wnt and Vg1 signalling pathways initiate primitive streak formation in the chick embryo. Development.

[bb0605] Skromne I., Stern C.D. (2002). A hierarchy of gene expression accompanying induction of the primitive streak by Vg1 in the chick embryo. Mech. Dev..

[bb0610] Song J., McColl J., Camp E., Kennerley N., Mok G.F., McCormick D., Grocott T., Wheeler G.N., Münsterberg A.E. (2014). Smad1 transcription factor integrates BMP2 and Wnt3a signals in migrating cardiac progenitor cells. Proc. Natl. Acad. Sci..

[bb0615] Spratt N.T., Haas H. (1960). Integrative mechanisms in development of the early chick blastoderm. 1. Regulative potentiality of separated parts. J. Exp. Zool..

[bb0620] Spratt N.T. (1942). Location of organ-specific regions and their relationship to the development of the primitive streak in the early chick blastoderm. J. Exp. Zool..

[bb0625] Spratt N.T. (1946). Formation of the primitive streak in the explanted chick blastoderm marked with carbon particles. J. Exp. Zool..

[bb0630] Stebler J., Spieler D., Slanchev K., Molyneaux K.A., Richter U., Cojocaru V., Tarabykin V., Wylie C., Kessel M., Raz E. (2004). Primordial germ cell migration in the chick and mouse embryo: the role of the chemokine SDF-1/CXCL12. Dev. Biol..

[bb0635] Stern C.D., Canning D.R. (1990). Origin of cells giving rise to mesoderm and endoderm in chick embryo. Nature.

[bb0640] Stolte D., Huang R., Christ B. (2002). Spatial and temporal pattern of Fgf-8 expression during chicken development. Anat. Embryol..

[bb0645] Stower M.J., Bertocchini F. (2017). The evolution of amniote gastrulation: the blastopore-primitive streak transition. Wiley Interdiscip. Rev. Dev. Biol..

[bb0650] Streit A., Lee K.J., Woo I., Roberts C., Jessell T.M., Stern C.D. (1998). Chordin regulates primitive streak development and the stability of induced neural cells, but is not sufficient for neural induction in the chick embryo. Development.

[bb0655] Sun Z., Amourda C., Shagirov M., Hara Y., Saunders Timothy E., Toyama Y. (2017). Basolateral protrusion and apical contraction cooperatively drive Drosophila germ-band extension. Nat. Cell Biol..

[bb0660] Sweetman D., Wagstaff L., Cooper O., Weijer C., Münsterberg A. (2008). The migration of paraxial and lateral plate mesoderm cells emerging from the late primitive streak is controlled by different Wnt signals. BMC Dev. Biol..

[bb0665] Tada M., Smith J.C. (2000). Xwnt11 is a target of Xenopus Brachyury: regulation of gastrulation movements via Dishevelled, but not through the canonical Wnt pathway. Development.

[bb0670] Torlopp A., Khan M.A.F., Oliveira N.M.M., Lekk I., Soto-Jiménez L.M., Sosinsky A., Stern C.D. (2014). The transcription factor Pitx2 positions the embryonic axis and regulates twinning. eLife.

[bb0675] Turner D.A., Girgin M., Alonso-Crisostomo L., Trivedi V., Baillie-Johnson P., Glodowski C.R., Hayward P.C., Collignon J., Gustavsen C., Serup P., Steventon B., P Lutolf M., Arias A.M. (2017). Anteroposterior polarity and elongation in the absence of extra-embryonic tissues and of spatially localised signalling in gastruloids: mammalian embryonic organoids. Development.

[bb0680] Vakaet L. (1962). Some new data concerning the formation of the definitive endoblast in the chick embryo. Development.

[bb0685] Vakaet L. (1984). The initiation of gastrular ingression in the chick blastoderm. Am. Zool..

[bb0690] van den Brink S.C., Baillie-Johnson P., Balayo T., Hadjantonakis A.K., Nowotschin S., Turner D.A., Martinez Arias A. (2014). Symmetry breaking, germ layer specification and axial organisation in aggregates of mouse embryonic stem cells. Development.

[bb0695] Vanderleest T.E., Smits C.M., Xie Y., Jewett C.E., Blankenship J.T., Loerke D. (2018). Vertex sliding drives intercalation by radial coupling of adhesion and actomyosin networks during. Elife.

[bb0700] Vasiev B., Balter A., Chaplain M., Glazier J.A., Weijer C.J. (2010). Modeling gastrulation in the chick embryo: formation of the primitive streak. PLoS One.

[bb0705] Voiculescu O., Bertocchini F., Wolpert L., Keller R.E., Stern C.D. (2007). The amniote primitive streak is defined by epithelial cell intercalation before gastrulation. Nature.

[bb0710] Voiculescu O., Bodenstein L., Lau I.J., Stern C.D. (2014). Local cell interactions and self-amplifying individual cell ingression drive amniote gastrulation. eLife.

[bb0715] von Baer K.E. (1828). Entwicklungsgeschichte des Hunchens im Eie, Bonntraeger, Konigsberg.

[bb0720] Waddington C.H., Gray J. (1932). III. Experiments on the development of chick and duck embryos, cultivated *in vitro*. Philosophical Transactions of the Royal Society of London. Series B, Containing Papers of a Biological Character.

[bb0725] Wagstaff L.J., Bellett G., Mogensen M.M., Munsterberg A. (2008). Multicellular rosette formation during cell ingression in the avian primitive streak. Dev. Dyn..

[bb0730] Wallingford J.B., Rowning B.A., Vogeli K.M., Rothbächer U., Fraser S.E., Harland R.M. (2000). Dishevelled controls cell polarity during Xenopus gastrulation. Nature.

[bb0735] Wei Y., Mikawa T. (2000). Formation of the avian primitive streak from spatially restricted blastoderm: evidence for polarized cell division in the elongating streak. Development.

[bb0740] Weinberger C., Brick I. (1982). Primary hypoblast development in the chick: II. The role of cell division. Wilhelm Roux's archives of developmental biology.

[bb0745] Wetzel R. (1929). Untersuchungen am Hühnchen. Die Entwicklung des Keims während der ersten beiden Bruttage, Untersuchungen am Hühnchen.

[bb0750] Williams M., Burdsal C., Periasamy A., Lewandoski M., Sutherland A. (2012). Mouse primitive streak forms in situ by initiation of epithelial to mesenchymal transition without migration of a cell population. Dev. Dyn..

[bb0755] Williams M., Yen W., Lu X., Sutherland A. (2014). Distinct apical and basolateral mechanisms drive planar cell polarity-dependent convergent extension of the mouse neural plate. Dev. Cell.

[bb0760] Woodcock M.E., Gheyas A.A., Mason A.S., Nandi S., Taylor L., Sherman A., Smith J., Burt D.W., Hawken R., McGrew M.J. (2019). Reviving rare chicken breeds using genetically engineered sterility in surrogate host birds. Proc. Natl. Acad. Sci..

[bb0765] Yamamoto M., Saijoh Y., Perea-Gomez A., Shawlot W., Behringer R.R., Ang S.L., Hamada H., Meno C. (2004). Nodal antagonists regulate formation of the anteroposterior axis of the mouse embryo. Nature.

[bb0770] Yanagawa N., Sakabe M., Sakata H., Yamagishi T., Nakajima Y. (2011). Nodal signal is required for morphogenetic movements of epiblast layer in the pre-streak chick blastoderm. Develop. Growth Differ..

[bb0775] Yang X., Dormann D., Munsterberg A.E., Weijer C.J. (2002). Cell movement patterns during gastrulation in the chick are controlled by positive and negative chemotaxis mediated by FGF4 and FGF8. Dev. Cell.

[bb0780] Yang X., Chrisman H., Weijer C.J. (2008). PDGF signalling controls the migration of mesoderm cells during chick gastrulation by regulating N-cadherin expression. Development.

[bb0785] Yu J.C., Fernandez-Gonzalez R. (2016). Local mechanical forces promote polarized junctional assembly and axis elongation in Drosophila. Elife.

[bb0790] Yue Q., Wagstaff L., Yang X., Weijer C., Munsterberg A. (2008). Wnt3a-mediated chemorepulsion controls movement patterns of cardiac progenitors and requires RhoA function. Development.

[bb0795] Zahavi N., Reich V., Khaner O. (1998). High proliferation rate characterizes the site of axis formation in the avian blastula-stage embryo. The International journal of developmental biology.

[bb0800] Zamir E.A., Czirok A., Cui C., Little C.D., Rongish B.J. (2006). Mesodermal cell displacements during avian gastrulation are due to both individual cell-autonomous and convective tissue movements. Proc. Natl. Acad. Sci. U. S. A..

[bb0805] Zeng X.X., Wilm T.P., Sepich D.S., Solnica-Krezel L. (2007). Apelin and its receptor control heart field formation during zebrafish gastrulation. Dev. Cell.

